# Supplementary material for: Characterization of New Proteomic Biomarker Candidates in Mucopolysaccharidosis Type IVA
Source: Int J Mol Sci. 2020 Dec 28;22(1):226. doi: 10.3390/ijms22010226 (PMC7795692; doi:10.3390/ijms22010226)
Supplement: Supplementary file 1 [file ijms-22-00226-s001.pdf]

International Journal of
Molecular Sciences

MDPI

Table S1. Protein List of Venn Diagram from Leukocyte Proteomic Study.

Combination Groups	Total Proteins	Code Prot.	Protein
CG, UG, ERT-a, ERT-b	139	P09211	Glutathione *S*-transferase P
		P00918	Carbonic anhydrase 2
		P13224	Platelet glycoprotein Ib beta chain
		P30101	Protein disulfide-isomerase A3
		P02675	Fibrinogen beta chain
		O15145	Actin-related protein 2/3 complex subunit 3
		P01859	Immunoglobulin heavy constant gamma 2
		O14773	Tripeptidyl-peptidase 1
		Q15404	Ras suppressor protein 1
		P06753	Tropomyosin alpha-3 chain
		P07996	Thrombospondin-1
		P04264	Keratin, type II cytoskeletal 1
		Q9ULV4	Coronin-1C
		P02647	Apolipoprotein A-I
		P59998	Actin-related protein 2/3 complex subunit 4
		O75594	Peptidoglycan recognition protein 1
		P32119	Peroxiredoxin-2
		P02775	Platelet basic protein
		P30041	Peroxiredoxin-6
		P11142	Heat shock cognate 71 kDa protein
		P63104	14-3-3 protein zeta/delta
		P22626	Heterogeneous nuclear ribonucleoproteins A2/B1
		P01876	Immunoglobulin heavy constant alpha 1
		O00194	Ras-related protein Rab-27B
		P01023	Alpha-2-macroglobulin
		P04179	Superoxide dismutase [Mn], mitochondrial
		P02671	Fibrinogen alpha chain
		P61158	Actin-related protein 3
		P04040	Catalase
		P37837	Transaldolase
		P63261	Actin, cytoplasmic 2
		P01871	Immunoglobulin heavy constant mu
		P62258	14-3-3 protein epsilon
		Q86UX7	Fermitin family homolog 3
		P06744	Glucose-6-phosphate isomerase
		P35908	Keratin, type II cytoskeletal 2 epidermal
		P14625	Endoplasmin
		P17213	Bactericidal permeability-increasing protein
		P04899	Guanine nucleotide-binding protein G(i) subunit alpha-2
		O75390	Citrate synthase, mitochondrial
		Q15907	Ras-related protein Rab-11B
		P02766	Transthyretin
		P62937	Peptidyl-prolyl cis-trans isomerase A
		P40197	Platelet glycoprotein V
		P14780	Matrix metalloproteinase-9

P01011	Alpha-1-antichymotrypsin
P11279	Lysosome-associated membrane glycoprotein 1
P99999	Cytochrome c
P08246	Neutrophil elastase
P61026	Ras-related protein Rab-10
P20700	Lamin-B1
Q01518	Adenylyl cyclase-associated protein 1
Q9HBI1	Beta-parvin
P07737	Profilin-1
P51149	Ras-related protein Rab-7a
Q06830	Peroxiredoxin-1
Q05315	Galectin-10
P02042	Hemoglobin subunit delta
P07359	Platelet glycoprotein Ib alpha chain
P29401	Transketolase
P11215	Integrin alpha-M
P01834	Immunoglobulin kappa constant
P02652	Apolipoprotein A-II
P05164	Myeloperoxidase
P61626	Lysozyme C
P30048	Thioredoxin-dependent peroxide reductase, mitochondrial
P30740	Leukocyte elastase inhibitor
P13645	Keratin, type I cytoskeletal 10
Q9Y490	Talin-1
P60660	Myosin light polypeptide 6
P0DMV9	Heat shock 70 kDa protein 1B
P31946	14-3-3 protein beta/alpha
P61769	Beta-2-microglobulin
Q13201	Multimerin-1
O15511	Actin-related protein 2/3 complex subunit 5
P01857	Immunoglobulin heavy constant gamma 1
P08670	Vimentin
P61224	Ras-related protein Rap-1b
P27695	DNA-(apurinic or apyrimidinic site) lyase
P02776	Platelet factor 4
P16671	Platelet glycoprotein 4
P02549	Spectrin alpha chain, erythrocytic 1
Q9H299	SH3 domain-binding glutamic acid-rich-like protein 3
P01619	Immunoglobulin kappa variable 3-20
P08514	Integrin alpha-IIb
P68871	Hemoglobin subunit beta
P04075	Fructose-bisphosphate aldolase A
P49913	Cathelicidin antimicrobial peptide
P60174	Triosephosphate isomerase
P19105	Myosin regulatory light chain 12A
P24158	Myeloblastin
P37802	Transgelin-2
P23528	Cofilin-1
P00738	Haptoglobin

		O75083	WD repeat-containing protein 1
		P06733	Alpha-enolase
		P62987	Ubiquitin-60S ribosomal protein L40
		P11021	Endoplasmic reticulum chaperone BiP
		P08962	CD63 antigen
		P06396	Gelsolin
		P21333	Filamin-A
		P06702	Protein S100-A9
		P10909	Clusterin
		P62805	Histone H4
		P07355	Annexin A2
		P23284	Peptidyl-prolyl cis-trans isomerase B
		P05106	Integrin beta-3
		P08575	Receptor-type tyrosine-protein phosphatase C
		P18206	Vinculin
		P22894	Neutrophil collagenase
		P35579	Myosin-9
		P02768	Serum albumin
		P07237	Protein disulfide-isomerase
		P61106	Ras-related protein Rab-14
		P13796	Plastin-2
		P01009	Alpha-1-antitrypsin
		P80188	Neutrophil gelatinase-associated lipocalin
		P02679	Fibrinogen gamma chain
		P05556	Integrin beta-1
		P02788	Lactotransferrin
		O15144	Actin-related protein 2/3 complex subunit 2
		P12814	Alpha-actinin-1
		P14770	Platelet glycoprotein IX
		P02763	Alpha-1-acid glycoprotein 1
		P27797	Calreticulin
		P04406	Glyceraldehyde-3-phosphate dehydrogenase
		P20160	Azurocidin
		P47756	F-actin-capping protein subunit beta
		P02730	Band 3 anion transport protein
		Q15084	Protein disulfide-isomerase A6
		P27105	Erythrocyte band 7 integral membrane protein
		P00915	Carbonic anhydrase 1
		P69905	Hemoglobin subunit alpha
		P08311	Cathepsin G
		P08567	Pleckstrin
		P35527	Keratin, type I cytoskeletal 9
		P05109	Protein S100-A8
		P26038	Moesin
		P06576	ATP synthase subunit beta, mitochondrial
CG, UG, ERT-a	8	P08758	Annexin A5
		P40926	Malate dehydrogenase, mitochondrial
		Q08722	Leukocyte surface antigen CD47
		P61160	Actin-related protein 2
		P69892	Hemoglobin subunit gamma-2
		P09622	Dihydrolipoyl dehydrogenase, mitochondrial

		P38646	Stress-70 protein, mitochondrial
		P11166	Solute carrier family 2, facilitated glucose transporter member 1
CG, UG, ERT-b	1	P61604	10 kDa heat shock protein, mitochondrial
UG, ERT-a, ERT-b	35	Q9BSJ8	Extended synaptotagmin-1
		Q9HDC9	Adipocyte plasma membrane-associated protein
		Q6UX06	Olfactomedin-4
		P11678	Eosinophil peroxidase
		P05107	Integrin beta-2
		P10599	Thioredoxin
		P54819	Adenylate kinase 2, mitochondrial
		P13727	Bone marrow proteoglycan
		P00387	NADH-cytochrome b5 reductase 3
		O43707	Alpha-actinin-4
		P46940	Ras GTPase-activating-like protein IQGAP1
		Q10588	ADP-ribosyl cyclase/cyclic ADP-ribose hydrolase 2
		P61978	Heterogeneous nuclear ribonucleoprotein K
		P52566	Rho GDP-dissociation inhibitor 2
		P01024	Complement C3
		P10153	Non-secretory ribonuclease
		P16284	Platelet endothelial cell adhesion molecule
		Q9Y624	Junctional adhesion molecule A
		P61981	14-3-3 protein gamma
		B9A064	Immunoglobulin lambda-like polypeptide 5
		Q14019	Coactosin-like protein
		P16109	P-selectin
		O75367	Core histone macro-H2A.1
		P01700	Immunoglobulin lambda variable 1-47
		P50995	Annexin A11
		P02787	Serotransferrin
		P52907	F-actin-capping protein subunit alpha-1
		P21926	CD9 antigen
		Q9P0L0	Vesicle-associated membrane protein-associated protein A
		P02749	Beta-2-glycoprotein 1
		P12724	Eosinophil cationic protein
		P41218	Myeloid cell nuclear differentiation antigen
		P50395	Rab GDP dissociation inhibitor beta
		P04275	von Willebrand factor
		Q6EEV6	Small ubiquitin-related modifier 4
CG, ERT-a, ERT-b	15	P14618	Pyruvate kinase PKM
		P12429	Annexin A3
		P05089	Arginase-1
		P04083	Annexin A1
		O00299	Chloride intracellular channel protein 1
		P07910	Heterogeneous nuclear ribonucleoproteins C1/C2
		P50552	Vasodilator-stimulated phosphoprotein
		P06312	Immunoglobulin kappa variable 4-1

		O15143	Actin-related protein 2/3 complex subunit 1B
		P52209	6-phosphogluconate dehydrogenase, decarboxylating
		P08133	Annexin A6
		P26447	Protein S100-A4
		P80511	Protein S100-A12
		P36957	Dihydrolipoyllysine-residue succinyltransferase component of 2-oxoglutarate dehydrogenase complex, mitochondrial
		P11277	Spectrin beta chain, erythrocytic
CG, UG	2	Q9BTM1	Histone H2A.J
		Q6P4A8	Phospholipase B-like 1
UG, ERT-a	27	P10619	Lysosomal protective protein
		Q96C19	EF-hand domain-containing protein D2
		P51659	Peroxisomal multifunctional enzyme type 2
		P02751	Fibronectin
		P55145	Mesencephalic astrocyte-derived neurotrophic factor
		P28066	Proteasome subunit alpha type-5
		P23368	NAD-dependent malic enzyme, mitochondrial
		P19367	Hexokinase-1
		P60953	Cell division control protein 42 homolog
		P07339	Cathepsin D
		Q9HD89	Resistin
		P04844	Dolichyl-diphosphooligosaccharide--protein glycosyltransferase subunit 2
		Q9H4B7	Tubulin beta-1 chain
		O75636	Ficolin-3
		Q13011	Delta(3,5)-Delta(2,4)-dienoyl-CoA isomerase, mitochondrial
		O94919	Endonuclease domain-containing 1 protein
		Q04917	14-3-3 protein eta
		P00491	Purine nucleoside phosphorylase
		P01137	Transforming growth factor beta-1 proprotein
		P50148	Guanine nucleotide-binding protein G(q) subunit alpha
		P13667	Protein disulfide-isomerase A4
		P00390	Glutathione reductase, mitochondrial
		Q13231	Chitotriosidase-1
		P49721	Proteasome subunit beta type-2
		P07900	Heat shock protein HSP 90-alpha
		P25787	Proteasome subunit alpha type-2
		Q9Y6C2	EMILIN-1
UG, ERT-b	3	P05155	Plasma protease C1 inhibitor
		P84077	ADP-ribosylation factor 1
		P01624	Immunoglobulin kappa variable 3-15
CG, ERT-a	12	P13647	Keratin, type II cytoskeletal 5
		Q92820	Gamma-glutamyl hydrolase
		Q5VTE0	Putative elongation factor 1-alpha-like 3
		P16157	Ankyrin-1
		P31146	Coronin-1 [a]

		P67936	Tropomyosin alpha-4 chain
		P61006	Ras-related protein Rab-8A
		Q14697	Neutral alpha-glucosidase AB
		P00488	Coagulation factor XIII A chain
		P00338	L-lactate dehydrogenase A chain
		P10809	60 kDa heat shock protein, mitochondrial
		Q99879	Histone H2B type 1-M
CG, ERT-b	3	P53597	Succinate--CoA ligase [ADP/GDP-forming] subunit alpha, mitochondrial
		P04433	Immunoglobulin kappa variable 3-11
		P0DOY3	Immunoglobulin lambda constant 3
ERT-a, ERT-b	16	P13639	Elongation factor 2
		Q9BQE3	Tubulin alpha-1C chain
		Q96AP7	Endothelial cell-selective adhesion molecule
		P27348	14-3-3 protein theta
		P17931	Galectin-3
		P27824	Calnexin
		P30040	Endoplasmic reticulum resident protein 29
		Q99439	Calponin-2
		P30044	Peroxiredoxin-5, mitochondrial
		P78417	Glutathione S-transferase omega-1
		P11169	Solute carrier family 2, facilitated glucose transporter member 3
		Q9Y2Y8	Proteoglycan 3
		P59666	Neutrophil defensin 3
		P31949	Protein S100-A11
		P13473	Lysosome-associated membrane glycoprotein 2
		O75015	Low affinity immunoglobulin gamma Fc region receptor III-B
UG	20	P28065	Proteasome subunit beta type-9
		P26641	Elongation factor 1-gamma
		P02774	Vitamin D-binding protein
		P40121	Macrophage-capping protein
		P25788	Proteasome subunit alpha type-3
		P32942	Intercellular adhesion molecule 3
		P36222	Chitinase-3-like protein 1
		O14672	Disintegrin and metalloproteinase domain-containing protein 10
		P62314	Small nuclear ribonucleoprotein Sm D1
		P04217	Alpha-1B-glycoprotein
		Q9NRW1	Ras-related protein Rab-6B
		P07437	Tubulin beta chain
		Q99880	Histone H2B type 1-L
		Q9NQC3	Reticulon-4
		P20292	Arachidonate 5-lipoxygenase-activating protein
		P20061	Transcobalamin-1
		P18669	Phosphoglycerate mutase 1
		P24557	Thromboxane-A synthase
		Q16778	Histone H2B type 2-E
		P42785	Lysosomal Pro-X carboxypeptidase

		P48735	Isocitrate dehydrogenase [NADP], mitochondrial
		P48059	LIM and senescent cell antigen-like-containing domain protein 1
		Q14677	Clathrin interactor 1
		P00558	Phosphoglycerate kinase 1
		O94925	Glutaminase kidney isoform, mitochondrial
		P16452	Erythrocyte membrane protein band 4.2
CG	15	P62879	Guanine nucleotide-binding protein G(I)/G(S)/G(T) subunit beta-2
		P26583	High mobility group protein B2
		P69891	Hemoglobin subunit gamma-1
		P02533	Keratin, type I cytoskeletal 14
		P06737	Glycogen phosphorylase, liver form
		P25705	ATP synthase subunit alpha, mitochondrial
		P55072	Transitional endoplasmic reticulum ATPase
		P60709	Actin, cytoplasmic 1
		Q562R1	Beta-actin-like protein 2
ERT-a.	49	P09651	Heterogeneous nuclear ribonucleoprotein A1
		P17301	Integrin alpha-2
		P68431	Histone H3.1
		Q9ULZ3	Apoptosis-associated speck-like protein containing a CARD
		Q01082	Spectrin beta chain, non-erythrocytic 1
		Q13813	Spectrin alpha chain, non-erythrocytic 1
		O75563	Src kinase-associated phosphoprotein 2
		P47755	F-actin-capping protein subunit alpha-2
		P51148	Ras-related protein Rab-5C
		P07225	Vitamin K-dependent protein S
		Q9HCN6	Platelet glycoprotein VI
		Q9UIB8	SLAM family member 5
		Q9UBW5	Bridging integrator 2
		Q16698	2,4-dienoyl-CoA reductase, mitochondrial
		Q96E39	RNA binding motif protein, X-linked-like-1
		P12259	Coagulation factor V
		O95831	Apoptosis-inducing factor 1, mitochondrial
		P0C0L5	Complement C4-B
		P11413	Glucose-6-phosphate 1-dehydrogenase
		P09467	Fructose-1,6-bisphosphatase 1
		Q5TFQ8	Signal-regulatory protein beta-1 isoform 3
		P68036	Ubiquitin-conjugating enzyme E2 L3
		P84243	Histone H3.3
		P40925	Malate dehydrogenase, cytoplasmic
		Q13418	Integrin-linked protein kinase
		P13598	Intercellular adhesion molecule 2
		P49720	Proteasome subunit beta type-3
		Q96AG4	Leucine-rich repeat-containing protein 59
		O75368	SH3 domain-binding glutamic acid-rich-like protein
		Q9UL25	Ras-related protein Rab-21
		O75629	rotein CREG1

		Q92882	Osteoclast-stimulating factor 1
		P07384	Calpain-1 catalytic subunit
		P63162	Small nuclear ribonucleoprotein-associated protein N
		Q14165	Malectin
		P52565	Rho GDP-dissociation inhibitor 1
		Q9Y3D6	Mitochondrial fission 1 protein
		P12931	Proto-oncogene tyrosine-protein kinase Src
		Q9P035	Very-long-chain (3R)-3-hydroxyacyl-CoA dehydratase 3
		Q9H0U4	Ras-related protein Rab-1B
		P61225	Ras-related protein Rap-2b
		Q9UFN0	Protein NipSnap homolog 3A
		Q99497	Parkinson disease protein 7
		P30405	Peptidyl-prolyl cis-trans isomerase F, mitochondrial
		P28676	Grancalcin
		O75695	Protein XRP2
		P29350	Tyrosine-protein phosphatase non-receptor type 6
		Q00839	Heterogeneous nuclear ribonucleoprotein U
		Q8NBM8	Prenylcysteine oxidase-like
ERT-b	10	Q71DI3	Histone H3.2
		P80748	Immunoglobulin lambda variable 3-21
		P00751	Complement factor B
		A2NJV5	Immunoglobulin kappa variable 2-29
		O75131	Copine-3
		P08637	Low affinity immunoglobulin gamma Fc region receptor III-A
		Q12913	Receptor-type tyrosine-protein phosphatase eta
		Q5SQ64	Lymphocyte antigen 6 complex locus protein G6f
		Q9H4G4	Golgi-associated plant pathogenesis-related protein 1
		Q9UQ80	Proliferation-associated protein 2G4

Table S2. SWATCH Study Protein Lists in Leukocyte Cells.

Upregulated Proteins in Control, Compared in Untreated			
**Protein**	**Group**	***p*-Value**	**FC**
P14618	Pyruvate kinase PKM	0,0079	18,6354
P04406	Glyceraldehyde-3-phosphate dehydrogenase	0,0003	18,5390
P00338	L-lactate dehydrogenase A chain	0,0023	15,8861
P16402	Histone H1.3	0,0007	13,1325
P16401	Histone H1.5	0,0030	11,1611
P00558	Phosphoglycerate kinase 1	0,0052	11,0840
Q9NTK5	Obg-like ATPase 1	0,0054	9,0976
P52209	6-phosphogluconate dehydrogenase, decarboxylating	0,0378	8,8029
P06737	Glycogen phosphorylase, liver form	0,0019	8,5721
P62826	GTP-binding nuclear protein Ran	0,0126	7,8279
P26038	Moesin	0,0018	7,8019
P31146	Coronin-1A	0,0268	7,7937
P06744	Glucose-6-phosphate isomerase	0,0182	6,0802
P26583	High mobility group protein B2	0,0043	5,3391
P10809	60 kDa heat shock protein, mitochondrial	0,0000	5,2493
P04083	Annexin A1	0,0018	4,9757
P68871	Hemoglobin subunit beta	0,0031	4,8483
P07195	L-lactate dehydrogenase B chain	0,0002	4,4237
P55072	Transitional endoplasmic reticulum ATPase	0,0000	4,2616
P30566	Adenylosuccinate lyase	0,0007	4,2306
P13804	Electron transfer flavoprotein subunit alpha, mitochondrial	0,0000	4,0450
P19971	Thymidine phosphorylase	0,0006	3,9674
P40199	Carcinoembryonic antigen-related cell adhesion molecule 6	0,0433	3,9420
O00299	Chloride intracellular channel protein 1	0,0006	3,9351
P07741	Adenine phosphoribosyltransferase	0,0219	3,7614
P69905	Hemoglobin subunit alpha	0,0146	3,6070
P05089	Arginase-1 OS=Homo sapiens	0,0134	3,5286
P17612	cAMP-dependent protein kinase catalytic subunit alpha	0,0112	3,4230
P35908	Keratin, type II cytoskeletal 2 epidermal	0,0106	3,4012
Q92882	Osteoclast-stimulating factor 1	0,0245	3,3752
P00488	Coagulation factor XIII A chain	0,0121	3,3421
P09493	Tropomyosin alpha-1 chain	0,0007	3,3362
P11177	Pyruvate dehydrogenase E1 component subunit beta, mitochondrial	0,0003	3,3274
P60709	Actin, cytoplasmic 1	0,0002	3,3227
P35527	Keratin, type I cytoskeletal 9	0,0111	3,3122
Q06323	Proteasome activator complex subunit 1	0,0021	3,2949
P25705	ATP synthase subunit alpha, mitochondrial	0,0002	3,2681
P31948	Stress-induced-phosphoprotein 1	0,0332	3,2562
O15143	Actin-related protein 2/3 complex subunit 1B	0,0333	3,1931
P36871	Phosphoglucomutase-1	0,0010	3,0452
P04264	Keratin, type II cytoskeletal 1 OS=Homo sapiens	0,0439	3,0298
P38606	V-type proton ATPase catalytic subunit A	0,0014	2,9531

P29401	Transketolase	0,0453	2,9138
Q5VTE0	Putative elongation factor 1-alpha-like 3	0,0017	2,8852
Q7L5Y6	DET1 homolog	0,0387	2,8429
P60981	Destrin	0,0126	2,7749
P61160	Actin-related protein 2	0,0126	2,7716
P68036	Ubiquitin-conjugating enzyme E2 L3	0,0011	2,7056
P02652	Apolipoprotein A-II	0,0362	2,7056
P61604	10 kDa heat shock protein, mitochondrial	0,0005	2,6459
P17931	Galectin-3	0,0223	2,5621
P11413	Glucose-6-phosphate 1-dehydrogenase	0,0436	2,5312
Q16181	Septin-7	0,0060	2,5013
P04075	Fructose-bisphosphate aldolase A	0,0022	2,4924
Q15833	Syntaxin-binding protein 2	0,0036	2,4736
O15511	Actin-related protein 2/3 complex subunit 5	0,0021	2,4691
Q96QK1	Vacuolar protein sorting-associated protein 35	0,0221	2,4674
P02538	Keratin, type II cytoskeletal 6A	0,0394	2,2979
P55786	Puromycin-sensitive aminopeptidase	0,0404	2,2783
Q00013	55 kDa erythrocyte membrane protein	0,0082	2,2653
P08571	Monocyte differentiation antigen CD14	0,0425	2,2551
P30044	Peroxiredoxin-5, mitochondrial	0,0250	2,2293
P35612	Beta-adducin	0,0446	2,2199
Q15366	Poly(rC)-binding protein 2	0,0314	2,1815
P67936	Tropomyosin alpha-4 chain	0,0291	2,1731
P09525	Annexin A4	0,0017	2,1524
P02766	Transthyretin	0,0349	2,0798
P50552	Vasodilator-stimulated phosphoprotein	0,0018	2,0793
P06576	ATP synthase subunit beta, mitochondrial	0,0005	2,0450
P08133	Annexin A6	0,0057	2,0414
Q16762	Thiosulfate sulfurtransferase	0,0145	2,0310
P61978	Heterogeneous nuclear ribonucleoprotein K	0,0046	1,9658
O15145	Actin-related protein 2/3 complex subunit 3	0,0355	1,9633
Q99798	Aconitate hydratase, mitochondrial	0,0010	1,9608
P01860	Immunoglobulin heavy constant gamma 3	0,0162	1,9486
P02533	Keratin, type I cytoskeletal 14	0,0475	1,9281
P30086	Phosphatidylethanolamine-binding protein	0,0357	1,8783
Q14697	Neutral alpha-glucosidase AB	0,0060	1,8336
Q9H2U2	Inorganic pyrophosphatase 2, mitocondrial	0,0006	1,8073
P30101	Protein disulfide-isomerase A3	0,0254	1,7864
Q99439	Calponin-2	0,0175	1,7689
P62136	Serine/threonine-protein phosphatase PP1-alpha catalytic subunit	0,0012	1,7576
P00505	Aspartate aminotransferase, mitochondrial	0,0139	1,7565
P30040	Endoplasmic reticulum resident protein 29	0,0135	1,7389
P09651	Heterogeneous nuclear ribonucleoprotein A1	0,0379	1,7249
P30049	ATP synthase subunit delta, mitochondrial	0,0068	1,6931
O75083	WD repeat-containing protein 1	0,0440	1,6445
Q13813	Spectrin alpha chain, non-erythrocytic 1	0,0301	1,6070
Q13492	Phosphatidylinositol-binding clathrin assembly protein	0,0096	1,5878
P40926	Malate dehydrogenase, mitochondrial	0,0049	1,4994
O75390	Citrate synthase, mitochondrial	0,0118	1,4719

Upregulated Proteins in Untreated, Compared in Control			
Protein	Group	*p*-Value	FC
O14773	Tripeptidyl-peptidase 1	0,0362	1,4441
Q15084	Protein disulfide-isomerase A6	0,0251	1,4635
P08567	Pleckstrin	0,0376	1,4669
P20338	Ras-related protein Rab-4A	0,0195	1,5322
P62258	14-3-3 protein epsilon	0,0110	1,5330
P63104	14-3-3 protein zeta/delta	0,0160	1,5440
Q8NBS9	Thioredoxin domain-containing protein 5	0,0446	1,5722
P04839	Cytochrome b-245 heavy chain	0,0475	1,5946
P61981	14-3-3 protein gamma	0,0085	1,6066
Q9HDC9	Adipocyte plasma membrane-associated protein	0,0235	1,6150
P61769	Beta-2-microglobulin	0,0439	1,6224
Q9UFN0	Protein NipSnap homolog 3ª	0,0224	1,6409
P05106	Integrin beta-3	0,0341	1,6777
P54578	Ubiquitin carboxyl-terminal hydrolase 14	0,0446	1,6928
P54108	Cysteine-rich secretory protein 3	0,0392	1,7124
P24557	Thromboxane-A synthase	0,0274	1,7477
P21333	Filamin-A	0,0365	1,7481
Q99623	Prohibitin-2	0,0165	1,7628
O94919	Endonuclease domain-containing 1 protein	0,0432	1,7709
P20061	Transcobalamin-1	0,0364	1,7894
P04844	Dolichyl-diphosphooligosaccharide--protein glycosyltransferase subunit 2	0,0017	1,8642
P30405	Peptidyl-prolyl cis-trans isomerase F, mitochondrial	0,0237	1,8656
P23229	Integrin alpha-6	0,0295	1,8854
P80188	Neutrophil gelatinase-associated lipocalin	0,0401	1,9830
P21926	CD9 antigen	0,0160	1,9864
Q9NQC3	Reticulon-4	0,0221	1,9867
P11234	Ras-related protein Ral-B	0,0252	2,0040
P24158	Myeloblastin	0,0269	2,0115
P07996	Thrombospondin-1	0,0014	2,0331
Q96P48	Arf-GAP with Rho-GAP domain, ANK repeat and PH domain-containing protein 1	0,0092	2,0360
P50990	T-complex protein 1 subunit theta	0,0170	2,0416
Q6UX71	Plexin domain-containing protein 2	0,0144	2,0905
P16109	P-selectin	0,0134	2,1039
Q5SQ64	Lymphocyte antigen 6 complex locus protein G6f	0,0161	2,1107
P62318	Small nuclear ribonucleoprotein Sm D3	0,0488	2,1112
Q13231	Chitotriosidase-1	0,0058	2,1411
Q14165	Malectin	0,0147	2,1586
P35579	Myosin-9	0,0084	2,1873
P51659	Peroxisomal multifunctional enzyme type 2	0,0015	2,2355
Q00325	Phosphate carrier protein, mitochondrial	0,0096	2,2758
P01137	Transforming growth factor beta-1 proprotein	0,0030	2,3360
P62314	Small nuclear ribonucleoprotein Sm D1	0,0015	2,3589
P12724	Eosinophil cationic protein	0,0012	2,4191

P02774	Vitamin D-binding protein	0,0439	2,4327
P00387	NADH-cytochrome b5 reductase 3	0,0015	2,4463
P12838	Neutrophil defensin 4	0,0091	2,4608
Q9BSJ8	Extended synaptotagmin-1	0,0004	2,4649
Q9Y6C2	EMILIN-1	0,0147	2,4765
Q9H4B7	Tubulin beta-1 chain	0,0344	2,6765
P61224	Ras-related protein Rap-1b	0,0006	2,8033
Q6UX06	Olfactomedin-4	0,0139	2,8132
P08246	Neutrophil elastase	0,0316	2,9474
Q8TC12	Retinol dehydrogenase 11	0,0422	3,0610
P14780	Matrix metalloproteinase-9	0,0104	3,1051
P02775	Platelet basic protein	0,0026	3,1669
P41218	Myeloid cell nuclear differentiation antigen	0,0203	3,2656
P00747	Plasminogen	0,0232	3,3382
P17213	Bactericidal permeability-increasing protein	0,0423	3,3966
P41240	Tyrosine-protein kinase CSK	0,0013	3,4288
P23219	Prostaglandin G/H synthase 1	0,0005	3,6929
Q8NBM8	Prenylcysteine oxidase-like	0,0002	3,8514
P02776	Platelet factor 4	0,0011	3,9596
P04004	Vitronectin	0,0305	4,0358
Q8WWA1	Transmembrane protein 40	0,0006	4,9507
P02749	Beta-2-glycoprotein 1	0,0240	5,3740
P05164	Myeloperoxidase	0,0038	5,5110
P20160	Azurocidin	0,0066	7,2103
P02788	Lactotransferrin	0,0009	7,6776
Q6DRA6	Putative histone H2B type 2-D	0,0040	8,3208
Q9BTM1	Histone H2A.J	0,0138	8,8293
P59666	Neutrophil defensin 3	0,0095	8,9484
Q99879	Histone H2B type 1-M	0,0060	9,9891
P62805	Histone H4	0,0079	12,0044

**Upregulated Proteins in Untreated, Compared in ERT-a**			
**Protein**	**Group**	***p*-Value**	**FC**
A0A075B6S5	Immunoglobulin kappa variable 1-27	0,0096	17,9362
P59666	Neutrophil defensin 3	0,0431	3,7253
P23141	Liver carboxylesterase 1	0,0013	3,2258
P04196	Histidine-rich glycoprotein	0,0322	2,5794
Q00325	Phosphate carrier protein, mitochondrial	0,0124	2,5252
Q6UX06	Olfactomedin-4	0,0283	2,4717
P12838	Neutrophil defensin 4	0,0204	2,3711
P02788	Lactotransferrin	0,0388	2,2862
P40306	Proteasome subunit beta type-10	0,0135	2,2613
Q99623	Prohibitin-2	0,0100	2,1771
P20338	Ras-related protein Rab-4A	0,0038	2,1434
Q13231	Chitotriosidase-1	0,0219	2,0308
P00367	Glutamate dehydrogenase 1, mitochondrial	0,0151	2,0051
P36222	Chitinase-3-like protein 1	0,0209	1,9798
P62314	Small nuclear ribonucleoprotein Sm D1	0,0130	1,9495
P04843	Dolichyl-diphosphooligosaccharide--protein glycosyltransferase subunit 1	0,0394	1,9426

P00492	Hypoxanthine-guanine phosphoribosyltransferase	0,0310	1,9243
Q96P48	Arf-GAP with Rho-GAP domain, ANK repeat and PH domain-containing protein 1	0,0227	1,8559
P00387	NADH-cytochrome b5 reductase 3	0,0275	1,7933
P54108	Cysteine-rich secretory protein 3	0,0355	1,7709
P57088	Transmembrane protein 33	0,0237	1,7296
P30048	Thioredoxin-dependent peroxide reductase, mitochondrial	0,0259	1,5081

**Upregulated Proteins in ERT-a, Compared in Untreated**			
**Protein**	**Group**	***p*-Value**	**FC**
P04075	Fructose-bisphosphate aldolase A	0,0472	1,4848
Q13813	Spectrin alpha chain, non-erythrocytic 1	0,0188	1,5269
P00338	L-lactate dehydrogenase A chain	0,0305	1,6499
P68036	Ubiquitin-conjugating enzyme E2 L3	0,0339	1,7144
Q96AG4	Leucine-rich repeat-containing protein 59	0,0005	1,7575
P30101	Protein disulfide-isomerase A3	0,0216	1,8021
O00299	Chloride intracellular channel protein 1	0,0302	1,8891
Q99439	Calponin-2	0,0156	2,1441
Q12913	Receptor-type tyrosine-protein phosphatase eta	0,0015	2,2441
P10153	Non-secretory ribonuclease	0,0293	2,4845

**Upregulated Proteins in Untreated, Compared in ERT-b**			
**Protein**	**Group**	***p*-Value**	**FC**
P04004	Vitronectin	0,0458	4,1505
P49748	Very long-chain specific acyl-CoA dehydrogenase, mitochondrial	0,0473	3,9345
P41240	Tyrosine-protein kinase CSK	0,0021	3,5935
P00367	Glutamate dehydrogenase 1, mitochondrial	0,0110	2,0881
P32119	Peroxiredoxin-2	0,0318	1,7739
P23141	Liver carboxylesterase 1	0,0479	1,7552
P47755	F-actin-capping protein subunit alpha-2	0,0400	1,7545
Q02218	2-oxoglutarate dehydrogenase, mitocondrial	0,0242	1,6811
P30048	Thioredoxin-dependent peroxide reductase, mitochondrial	0,0255	1,5177
P11310	Medium-chain specific acyl-CoA dehydrogenase, mitochondrial	0,0186	1,4789

**Upregulated Proteins in ERT-b, Compared in Untreated**			
**Protein**	**Group**	***p*-Value**	**FC**
Q06323	Proteasome activator complex subunit 1	0,0500	1,4306
P04075	Fructose-bisphosphate aldolase A	0,0220	1,5273
P31146	Coronin-1A	0,0195	1,5744
P06737	Glycogen phosphorylase, liver form	0,0114	1,7951
P27695	DNA-(apurinic or apyrimidinic site) lyase	0,0281	1,8630
P00338	L-lactate dehydrogenase A chain	0,0108	1,8679
P02042	Hemoglobin subunit delta	0,0138	1,8726

P68871	Hemoglobin subunit beta	0,0251	1,8887
P00558	Phosphoglycerate kinase 1	0,0456	1,9304
P52209	6-phosphogluconate dehydrogenase, decarboxylating	0,0130	1,9527
P19971	Thymidine phosphorylase	0,0358	1,9770
P08133	Annexin A6	0,0337	1,9848
P06744	Glucose-6-phosphate isomerase	0,0111	2,0416
P30101	Protein disulfide-isomerase A3	0,0295	2,1057
O43684	Mitotic checkpoint protein BUB3	0,0120	2,1731
P26038	Moesin	0,0338	2,2219
P14618	Pyruvate kinase PKM	0,0351	2,2541
O00299	Chloride intracellular channel protein 1	0,0157	2,4014
Q9Y2Y8	Proteoglycan 3	0,0116	2,6604
P26583	High mobility group protein B2	0,0150	3,3444
P04083	Annexin A1	0,0155	3,3598
P11678	Eosinophil peroxidase	0,0266	3,4018
P10153	Non-secretory ribonuclease	6,1420	6,1420

**Upregulated Proteins in ERT-a, Compared in ERT-b**			
**Protein**	**Group**	***p*-Value**	**FC**
Q9HCN6	Platelet glycoprotein VI	0,0141	2,1295
P22626	Heterogeneous nuclear ribonucleoproteins A2/B1	0,0478	1,7615
O75695	Protein XRP2	0,0426	1,3579
P62258	14-3-3 protein epsilon	0,0047	1,2943

**Upregulated proteins in ERT-b, compared in ERT-a**			
**Protein**	**Group**	***p*-Value**	**FC**
P06737	Glycogen phosphorylase, liver form	0,0429	1,4528
Q99798	Aconitate hydratase, mitochondrial	0,0319	1,6310
P50991	T-complex protein 1 subunit delta	0,0368	1,6979
O60496	Docking protein 2	0,0196	1,8275
P19971	Thymidine phosphorylase	0,0466	1,8415
P78371	T-complex protein 1 subunit beta	0,0495	1,8571
Q9NUJ1	Mycophenolic acid acyl-glucuronide esterase, mitochondrial	0,0353	1,9314
P04843	Dolichyl-diphosphooligosaccharide--protein glycosyltransferase subunit 1	0,0267	1,9449
P55072	Transitional endoplasmic reticulum ATPase	0,0493	1,9786
P20338	Ras-related protein Rab-4A	0,0299	2,0159
Q9NZN3	EH domain-containing protein 3	0,0321	2,0804
P41218	Myeloid cell nuclear differentiation antigen	0,0374	2,3092

**Upregulated Proteins in Control, Compared in ERT-a**			
**Protein**	**Group**	***p*-Value**	**FC**
P04406	Glyceraldehyde-3-phosphate dehydrogenase	0,0009	13,7240
P14618	Pyruvate kinase PKM	0,0171	12,9919
P00338	L-lactate dehydrogenase A chain	0,0066	9,6288
P00558	Phosphoglycerate kinase 1	0,0120	8,8984
P62826	GTP-binding nuclear protein Ran	0,0213	8,4856

P26038	Moesin	0,0042	7,7890
P30566	Adenylosuccinate lyase	0,0003	7,3462
P31146	Coronin-1A	0,0463	7,0536
P06737	Glycogen phosphorylase, liver form	0,0052	6,9374
P13804	Electron transfer flavoprotein subunit alpha, mitochondrial	0,0000	6,3910
P55072	Transitional endoplasmic reticulum ATPase	0,0000	5,0585
P07195	L-lactate dehydrogenase B chain	0,0004	4,8799
P60709	Actin, cytoplasmic 1	0,0001	4,6201
Q16181	Septin-7	0,0021	3,9887
P07741	Adenine phosphoribosyltransferase	0,0311	3,9742
P11177	Pyruvate dehydrogenase E1 component subunit beta, mitocondrial	0,0001	3,9682
P36871	Phosphoglucomutase-1	0,0009	3,9615
P19971	Thymidine phosphorylase	0,0014	3,6955
P60981	Destrin	0,0095	3,6291
P25705	ATP synthase subunit alpha, mitochondrial	0,0006	3,6018
P17612	cAMP-dependent protein kinase catalytic subunit alpha	0,0174	3,5329
P16402	Histone H1.3	0,0269	3,4692
P02652	Apolipoprotein A-II	0,0325	3,3625
P00488	Coagulation factor XIII A chain	0,0176	3,2396
Q15366	Poly(rC)-binding protein 2	0,0199	3,0474
P61160	Actin-related protein 2	0,0195	3,0163
P19827	Inter-alpha-trypsin inhibitor heavy chain H1	0,0242	2,9278
Q96QK1	Vacuolar protein sorting-associated protein 35	0,0205	2,8957
P61604	10 kDa heat shock protein, mitochondrial	0,0046	2,8276
P08571	Monocyte differentiation antigen CD14	0,0328	2,7823
Q96HE7	ERO1-like protein alpha	0,0469	2,7661
P61158	Actin-related protein 3	0,0414	2,7378
Q5VTE0	Putative elongation factor 1-alpha-like 3	0,0061	2,7129
P62937	Peptidyl-prolyl cis-trans isomerase A	0,0289	2,7129
P49748	Very long-chain specific acyl-CoA dehydrogenase, mitochondrial	0,0065	2,6313
P48735	Isocitrate dehydrogenase [NADP], mitochondrial	0,0036	2,5708
P38606	V-type proton ATPase catalytic subunit A	0,0076	2,4614
P80511	Protein S100-A12	0,0098	2,4223
Q99798	Aconitate hydratase, mitochondrial	0,0006	2,4216
P02766	Transthyretin	0,0362	2,4000
P00505	Aspartate aminotransferase, mitochondrial	0,0008	2,3502
Q00013	55 kDa erythrocyte membrane protein	0,0169	2,3394
Q14697	Neutral alpha-glucosidase AB	0,0022	2,2915
O15145	Actin-related protein 2/3 complex subunit 3	0,0307	2,2323
P62136	Serine/threonine-protein phosphatase PP1-alpha catalytic subunit	0,0088	2,1843
P10809	60 kDa heat shock protein, mitochondrial	0,0063	2,1241
O00299	Chloride intracellular channel protein 1	0,0132	2,0831
P01860	Immunoglobulin heavy constant gamma 3	0,0137	2,0003
O75083	WD repeat-containing protein 1	0,0172	1,9767
O15511	Actin-related protein 2/3 complex subunit 5	0,0164	1,9209

P48059	LIM and senescent cell antigen-like-containing domain protein 1	0,0383	1,9081
P01019	Angiotensinogen	0,0287	1,8872
Q15833	Syntaxin-binding protein 2	0,0118	1,8581
P40926	Malate dehydrogenase, mitochondrial	0,0014	1,8529
P00367	Glutamate dehydrogenase 1, mitochondrial	0,0250	1,8473
Q9NUJ1	Mycophenolic acid acyl-glucuronide esterase, mitochondrial	0,0432	1,8335
P61978	Heterogeneous nuclear ribonucleoprotein K	0,0109	1,7219
O75390	Citrate synthase, mitochondrial	0,0038	1,7114
P04075	Fructose-bisphosphate aldolase A	0,0284	1,6786
Q96C19	EF-hand domain-containing protein D2	0,0333	1,6613
P05107	Integrin beta-2	0,0229	1,5884
P09622	Dihydrolipoyl dehydrogenase, mitocondrial	0,0158	1,5506
Q9H2U2	Inorganic pyrophosphatase 2, mitochondrial	0,0159	1,5000
Q86UX7	Fermitin family homolog 3	0,0271	1,4976

**Upregulated Proteins in ERT-a, Compared in Control**			
**Protein**	**Group**	***p*-Value**	**FC**
P31946	14-3-3 protein beta/alpha	0,0125	1,3313
P04844	Dolichyl-diphosphooligosaccharide--protein glycosyltransferase subunit 2	0,0465	1,3673
Q6EEV6	Small ubiquitin-related modifier 4	0,0135	1,3945
Q15084	Protein disulfide-isomerase A6	0,0085	1,4089
P27348	14-3-3 protein theta	0,0028	1,4761
P19105	c regulatory light chain 12A	0,0149	1,4931
P61981	14-3-3 protein gamma	0,0065	1,5160
Q8NBS9	Thioredoxin domain-containing protein 5	0,0291	1,5313
P04179	Superoxide dismutase [Mn], mitocondrial	0,0012	1,5424
Q15907	Ras-related protein Rab-11B	0,0056	1,5674
P05106	Integrin beta-3	0,0160	1,5714
P05556	Integrin beta-1	0,0052	1,5743
P62258	14-3-3 protein epsilon	0,0000	1,5830
O95866	Megakaryocyte and platelet inhibitory receptor G6b	0,0208	1,7089
P40197	Platelet glycoprotein V	0,0080	1,7398
P14780	Matrix metalloproteinase-9	0,0303	1,7510
Q9HCN6	Platelet glycoprotein VI	0,0181	1,7887
P11234	Ras-related protein Ral-B	0,0062	1,7917
P51659	Peroxisomal multifunctional enzyme type 2	0,0002	1,8388
Q9BSJ8	Extended synaptotagmin-1	0,0064	1,9004
P63104	14-3-3 protein zeta/delta	0,0000	1,9036
P30405	Peptidyl-prolyl cis-trans isomerase F, mitochondrial	0,0035	1,9106
P54578	Ubiquitin carboxyl-terminal hydrolase 14	0,0138	1,9124
P07996	Thrombospondin-1	0,0174	1,9446
P21926	CD9 antigen	0,0016	1,9703
Q14165	Malectin	0,0003	1,9806
Q6UX71	Plexin domain-containing protein 2	0,0048	2,0308
P23229	Integrin alpha-6	0,0240	2,0577
O94919	Endonuclease domain-containing 1 protein	0,0005	2,1525

P10909	Clusterin	0,0421	2,2597
P07359	Platelet glycoprotein Ib alpha chain	0,0000	2,2665
P61224	Ras-related protein Rap-1b	0,0005	2,3011
P23219	Prostaglandin G/H synthase 1	0,0111	2,3568
P21333	Filamin-A	0,0010	2,3744
P01137	Transforming growth factor beta-1 proprotein	0,0000	2,3934
P59666	Neutrophil defensin 3	0,0390	2,4021
P12724	Eosinophil cationic protein	0,0193	2,4820
Q9Y490	Talin-1	0,0017	2,5597
Q0ZGT2	Nexilin	0,0256	2,5845
P16109	P-selectin	0,0010	2,6412
P05164	Myeloperoxidase	0,0266	2,7387
Q8NF91	Nesprin-1	0,0404	2,7787
Q9Y6C2	EMILIN-1	0,0004	2,8579
P02775	Platelet basic protein	0,0012	2,8929
P02776	Platelet factor 4	0,0081	2,8998
P10153	Non-secretory ribonuclease	0,0340	3,0428
Q8WWA1	Transmembrane protein 40	0,0266	3,1491
P02788	Lactotransferrin	0,0351	3,3583
P20160	Azurocidin	0,0362	3,5203
Q6DRA6	Putative histone H2B type 2-D	0,0149	3,5913
Q9BTM1	Histone H2A.J	0,0201	3,8062
P62805	Histone H4	0,0217	3,8947
Q8NBM8	Prenylcysteine oxidase-like	0,0038	4,1117
P13501	C-C motif chemokine 5	0,0107	4,6645
Q99879	Histone H2B type 1-M	0,0221	4,7259

**Upregulated Proteins in Control Group, Compared in ERT-b**			
**Protein**	**Group**	***p*-Value**	**FC**
P04406	Glyceraldehyde-3-phosphate dehydrogenase	0,0008	15,8202
P00338	L-lactate dehydrogenase A chain	0,0072	8,5049
P14618	Pyruvate kinase PKM	0,0217	8,2673
P13804	Electron transfer flavoprotein subunit alpha, mitochondrial	0,0000	6,4835
P00558	Phosphoglycerate kinase 1	0,0176	5,7420
P49748	Very long-chain specific acyl-CoA dehydrogenase, mitochondrial	0,0017	5,0070
P06737	Glycogen phosphorylase, liver form	0,0082	4,7752
Q9NTK5	Obg-like ATPase 1	0,0228	4,5744
P16402	Histone H1.3	0,0071	4,3911
P07195	L-lactate dehydrogenase B chain	0,0004	4,2405
P60709	Actin, cytoplasmic 1	0,0002	3,9994
P23528	Cofilin-1	0,0313	3,6348
P26038	Moesin	0,0143	3,5113
P00488	Coagulation factor XIII A chain	0,0149	3,2860
Q16181	Septin-7	0,0078	3,2061
P61604	10 kDa heat shock protein, mitochondrial	0,0007	3,1484
P62937	Peptidyl-prolyl cis-trans isomerase A	0,0199	3,1189
P11177	Pyruvate dehydrogenase E1 component subunit beta, mitochondrial	0,0006	3,0808

P48735	Isocitrate dehydrogenase [NADP], mitochondrial	0,0005	3,0282
P38606	V-type proton ATPase catalytic subunit A	0,0028	2,9313
P61160	Actin-related protein 2	0,0278	2,8040
P10809	60 kDa heat shock protein, mitochondrial	0,0003	2,7833
P68871	Hemoglobin subunit beta	0,0242	2,5670
P55072	Transitional endoplasmic reticulum ATPase	0,0003	2,5566
P00505	Aspartate aminotransferase, mitochondrial	0,0005	2,5442
Q5VTE0	Putative elongation factor 1-alpha-like 3	0,0140	2,3533
P60981	Destrin	0,0360	2,3203
Q06323	Proteasome activator complex subunit 1	0,0175	2,3031
Q14697	Neutral alpha-glucosidase AB	0,0018	2,1677
P01860	Immunoglobulin heavy constant gamma 3	0,0334	2,0441
O15511	Actin-related protein 2/3 complex subunit 5	0,0125	2,0411
O75083	WD repeat-containing protein 1	0,0092	2,0348
P19971	Thymidine phosphorylase	0,0195	2,0068
P00367	Glutamate dehydrogenase 1, mitochondrial	0,0179	1,9237
P25705	ATP synthase subunit alpha, mitochondrial	0,0423	1,9045
P68036	Ubiquitin-conjugating enzyme E2 L3	0,0303	1,9032
P62136	Serine/threonine-protein phosphatase PP1-alpha catalytic subunit	0,0036	1,6805
P04075	Fructose-bisphosphate aldolase A	0,0303	1,6319
Q15833	Syntaxin-binding protein 2	0,0471	1,6273
P30040	Endoplasmic reticulum resident protein 29	0,0238	1,6181
P61978	Heterogeneous nuclear ribonucleoprotein K	0,0233	1,5864
P40926	Malate dehydrogenase, mitochondrial	0,0097	1,5290
P50552	Vasodilator-stimulated phosphoprotein	0,0374	1,5177
O75390	Citrate synthase, mitochondrial	0,0262	1,5068
Q9NZ08	Endoplasmic reticulum aminopeptidase 1	0,0371	1,4936
Q99798	Aconitate hydratase, mitochondrial	0,0364	1,4848
Q06830	Peroxiredoxin-1	0,0207	1,4504
Q9P0L0	Vesicle-associated membrane protein-associated protein A	0,0302	1,3333
P38646	Stress-70 protein, mitochondrial	0,0469	1,3078

**Upregulated Proteins in ERT-b, Compared in Control**			
**Protein**	**Group**	***p*-Value**	**FC**
P62258	14-3-3 protein epsilon	0,0487	1,2231
Q15907	Ras-related protein Rab-11B	0,0097	1,4119
P04179	Superoxide dismutase [Mn], mitochondrial	0,0072	1,4411
P62987	Ubiquitin-60S ribosomal protein L40	0,0438	1,4438
P61981	14-3-3 protein gamma	0,0252	1,4442
P30405	Peptidyl-prolyl cis-trans isomerase F, mitochondrial	0,0414	1,4657
P63104	14-3-3 protein zeta/delta	0,0104	1,5148
P50148	Guanine nucleotide-binding protein G(q) subunit alpha	0,0430	1,5767
P08514	Integrin alpha-IIb	0,0477	1,5841
P50990	T-complex protein 1 subunit theta	0,0284	1,6454

P51659	Peroxisomal multifunctional enzyme type 2	0,0232	1,6741
P05556	Integrin beta-1	0,0403	1,7352
P07359	Platelet glycoprotein Ib alpha chain	0,0026	1,7877
P11234	Ras-related protein Ral-B	0,0083	1,8574
P05106	Integrin beta-3	0,0076	1,8584
Q9NQC3	Reticulon-4	0,0123	1,8796
P07996	Thrombospondin-1	0,0048	1,8981
P01137	Transforming growth factor beta-1 proprotein	0,0008	1,8996
Q6UX71	Plexin domain-containing protein 2	0,0108	1,9025
O95866	Megakaryocyte and platelet inhibitory receptor G6b	0,0330	1,9063
P00387	NADH-cytochrome b5 reductase 3	0,0341	1,9314
O94919	Endonuclease domain-containing 1 protein	0,0160	1,9710
P61769	Beta-2-microglobulin	0,0118	1,9734
P21926	CD9 antigen	0,0102	1,9927
P21333	Filamin-A	0,0099	2,0047
Q9BSJ8	Extended synaptotagmin-1	0,0183	2,0097
Q9Y6C2	EMILIN-1	0,0499	2,0530
Q5SQ64	Lymphocyte antigen 6 complex locus protein G6f	0,0008	2,0983
Q14165	Malectin	0,0175	2,2236
P00747	Plasminogen	0,0271	2,2546
P61224	Ras-related protein Rap-1b	0,0007	2,3479
P16109	P-selectin	0,0127	2,5707
Q9NTJ5	Phosphatidylinositol-3-phosphatase SAC1	0,0291	2,6961
P23229	Integrin alpha-6	0,0046	2,7222
P23219	Prostaglandin G/H synthase 1	0,0196	2,7377
P17213	Bactericidal permeability-increasing protein	0,0430	2,8220
Q15149	Plectin	0,0123	2,9715
P02775	Platelet basic protein	0,0000	3,0305
P02776	Platelet factor 4	0,0027	3,0395
P08246	Neutrophil elastase	0,0443	3,2181
Q8NBM8	Prenylcysteine oxidase-like	0,0002	3,4635
Q8TC12	Retinol dehydrogenase 11	0,0005	3,4661
P08311	Cathepsin G	0,0257	3,6636
Q8WWA1	Transmembrane protein 40	0,0122	4,1250
P05164	Myeloperoxidase	0,0185	4,2816
P12724	Eosinophil cationic protein	0,0027	4,3243
P41218	Myeloid cell nuclear differentiation antigen	0,0010	4,4056
P02788	Lactotransferrin	0,0208	5,3683
P11678	Eosinophil peroxidase	0,0127	5,5128
P62805	Histone H4	0,0235	5,7115
Q6DRA6	Putative histone H2B type 2-D	0,0033	6,1375
P20160	Azurocidin	0,0313	6,2332
Q9BTM1	Histone H2A.J	0,0029	6,5464
Q99879	Histone H2B type 1-M	0,0017	6,8123

P10153	Non-secretory ribonuclease	0,0402	7,5221
P13501	C-C motif chemokine 5	0,0000	8,0026

Table S3. P-Values of Downregulated proteins in untreated and ERT-treated MPS IVA patients (ERT-a, ERT-b) with respect to healthy controls.

UniProt Code	*p*-Value-UG	FC	1/FC	*p*-Value-ERT-a	FC	1/FC	*p*-Value-ERT-b	FC	1/FC
P14618	0,00790	18,6354	0,0537	0,01711	12,9919	0,0770	0,02175	8,26727	0,12096
P04406	0,00028	18,5390	0,0539	0,00092	13,7240	0,0729	0,00083	15,82022	0,06321
P00338	0,00227	15,8861	0,0629	0,00657	9,6288	0,1039	0,00716	8,50492	0,11758
P00558	0,00521	11,0840	0,0902	0,01197	8,8984	0,1124	0,01757	5,74196	0,17416
P07195	0,00020	4,4237	0,2261	0,00043	4,8799	0,2049	0,00040	4,24047	0,23582
P11177	0,00025	3,3274	0,3005	0,00007	3,9682	0,2520	0,00056	3,08079	0,32459
P04075	0,00215	2,4924	0,4012	0,02837	1,6786	0,5957	0,03026	1,63191	0,61278
P06744	0,01818	6,0802	0,1645	0,07907	2,9407	0,3401	0,07283	2,97811	0,33578
P36871	0,00102	3,0452	0,3284	0,00093	3,9615	0,2524	0,06494	1,71947	0,58157
P11413	0,04359	2,5312	0,3951	0,08456	2,1810	0,4585	0,15349	1,75005	0,57141
P26038	0,00183	7,8019	0,1282	0,00423	7,7890	0,1284	0,01426	3,51130	0,28479
P60981	0,01264	2,7749	0,3604	0,00949	3,6291	0,2756	0,03596	2,32032	0,43097
O15145	0,03550	1,9633	0,5094	0,03075	2,2323	0,4480	0,07432	1,89544	0,52758
P31146	0,02682	7,7937	0,1283	0,04627	7,0536	0,1418	0,06065	4,95010	0,20202
P09493	0,00067	3,3362	0,2997	0,17578	1,5345	0,6517	0,54669	1,26867	0,78822
P35527	0,01110	3,3122	0,3019	0,21018	1,6810	0,5949	0,39924	1,45839	0,68569
P35908	0,01057	3,4012	0,2940	0,22421	1,6262	0,6149	0,80451	1,15582	0,86519
O15143	0,03327	3,1931	0,3132	0,14805	2,2362	0,4472	0,08785	2,45594	0,40718
P04264	0,04388	3,0298	0,3301	0,31797	1,6514	0,6055	0,56492	1,35910	0,73578
P02538	0,03943	2,2979	0,4352	0,80581	0,9159	1,0918	0,39320	0,43651	2,29091
P67936	0,02908	2,1731	0,4602	0,72523	1,1393	0,8777	0,12007	1,81107	0,55216
P02533	0,04754	1,9281	0,5186	0,61264	1,2025	0,8316	0,54192	1,29002	0,77518
P61160	0,01259	2,7716	0,3608	0,01952	3,0163	0,3315	0,02779	2,80400	0,35663
O15511	0,00214	2,4691	0,4050	0,01635	1,9209	0,5206	0,01250	2,04112	0,48993
P60709	0,00017	3,3227	0,3010	0,00010	4,6201	0,2164	0,00019	3,99936	0,25004
Q99439	0,01753	1,7689	0,5653	0,31092	0,8250	1,2121	0,68624	1,09535	0,91295
P35612	0,04459	2,2199	0,4505	0,07243	1,8671	0,5356	0,54043	1,27007	0,78736
Q13813	0,03011	1,6070	0,6223	0,77730	1,0525	0,9501	0,76590	1,07432	0,93083
P50552	0,00181	2,0793	0,4809	0,07763	1,3778	0,7258	0,03742	1,51767	0,65890
O75083	0,04398	1,6445	0,6081	0,01725	1,9767	0,5059	0,00923	2,03483	0,49144
Q16181	0,00596	2,5013	0,3998	0,00211	3,9887	0,2507	0,00776	3,20606	0,31191
P17931	0,02231	2,5621	0,3903	0,07952	2,0125	0,4969	0,18135	1,63924	0,61004
Q96QK1	0,02213	2,4674	0,4053	0,02051	2,8957	0,3453	0,62150	1,27891	0,78192
Q15833	0,00364	2,4736	0,4043	0,01182	1,8581	0,5382	0,04713	1,62730	0,61451
P68036	0,00112	2,7056	0,3696	0,05808	1,5782	0,6336	0,03026	1,90323	0,52542
P30040	0,01351	1,7389	0,5751	0,32260	1,2186	0,8206	0,02382	1,61809	0,61801
Q13492	0,00960	1,5878	0,6298	0,05289	1,5561	0,6427	0,11727	1,43264	0,69801
P30049	0,00680	1,6931	0,5906	0,53776	1,1567	0,8645	0,74581	1,08226	0,92399
P25705	0,00023	3,2681	0,3060	0,00057	3,6018	0,2776	0,04230	1,90452	0,52507
O75390	0,01178	1,4719	0,6794	0,00385	1,7114	0,5843	0,02618	1,50682	0,66365
P30044	0,02502	2,2293	0,4486	0,12596	1,6549	0,6043	0,10413	1,83311	0,54552
P06576	0,00053	2,0450	0,4890	0,13495	1,3369	0,7480	0,12505	1,41316	0,70763
Q99798	0,00098	1,9608	0,5100	0,00059	2,4216	0,4129	0,03639	1,48480	0,67349
P13804	0,00001	4,0450	0,2472	0,00000	6,3910	0,1565	0,00000	6,48352	0,15424

P10809	0,00000	5,2493	0,1905	0,00629	2,1241	0,4708	0,00026	2,78327	0,35929
P61604	0,00053	2,6459	0,3779	0,00456	2,8276	0,3537	0,00069	3,14836	0,31763
P00505	0,01390	1,7565	0,5693	0,00082	2,3502	0,4255	0,00045	2,54421	0,39305
P40926	0,00487	1,4994	0,6669	0,00138	1,8529	0,5397	0,00973	1,52901	0,65402
P69905	0,01456	3,6070	0,2772	0,28477	1,5845	0,6311	0,06217	2,38018	0,42014
P68871	0,00312	4,8483	0,2063	0,35957	1,4975	0,6678	0,02418	2,56696	0,38957
P55072	0,00001	4,2616	0,2347	0,00001	5,0585	0,1977	0,00030	2,55665	0,39114
Q14697	0,00598	1,8336	0,5454	0,00223	2,2915	0,4364	0,00179	2,16773	0,46131
P16402	0,00069	13,1325	0,0761	0,02693	3,4692	0,2883	0,00713	4,39111	0,22773
P16401	0,00302	11,1611	0,0896	0,69273	1,3543	0,7384	0,93415	0,95581	1,04623
P08133	0,00566	2,0414	0,4899	0,10789	1,4113	0,7086	0,83936	1,02851	0,97228
P09525	0,00169	2,1524	0,4646	0,13379	1,4652	0,6825	0,14316	1,31800	0,75872
P04083	0,00182	4,9757	0,2010	0,09142	1,7698	0,5650	0,18913	1,48095	0,67524
P52209	0,03780	8,8029	0,1136	0,06949	6,3699	0,1570	0,08984	4,50808	0,22182
P05089	0,01343	3,5286	0,2834	0,05122	2,4578	0,4069	0,06146	2,26193	0,44210
P29401	0,04527	2,9138	0,3432	0,06156	2,9783	0,3358	0,05156	3,34076	0,29933
Q16762	0,01453	2,0310	0,4924	0,37557	1,2729	0,7856	0,50159	1,23741	0,80814
P30566	0,00070	4,2306	0,2364	0,00029	7,3462	0,1361	0,59558	1,35666	0,73711
Q00013	0,00815	2,2653	0,4414	0,01694	2,3394	0,4275	0,25515	1,59700	0,62617
P02766	0,03495	2,0798	0,4808	0,03616	2,4000	0,4167	0,14350	1,79447	0,55727
Q9H2U2	0,00061	1,8073	0,5533	0,01593	1,5000	0,6667	0,09855	1,35628	0,73731
Q7L5Y6	0,03865	2,8429	0,3518	0,13389	1,7815	0,5613	0,20109	1,58353	0,63150
P19971	0,00061	3,9674	0,2521	0,00143	3,6955	0,2706	0,01949	2,00677	0,49831
P00488	0,01213	3,3421	0,2992	0,01760	3,2396	0,3087	0,01486	3,28603	0,30432
P06737	0,00193	8,5721	0,1167	0,00523	6,9374	0,1441	0,00817	4,77521	0,20941
P62136	0,00119	1,7576	0,5690	0,00881	2,1843	0,4578	0,00363	1,68048	0,59507
P30101	0,02543	1,7864	0,5598	0,96899	0,9913	1,0088	0,54257	0,84835	1,17875
P55786	0,04041	2,2783	0,4389	0,16748	1,5985	0,6256	0,18618	1,52446	0,65597
P07741	0,02186	3,7614	0,2659	0,03114	3,9742	0,2516	0,06858	3,12402	0,32010
Q06323	0,00209	3,2949	0,3035	0,05653	2,1081	0,4744	0,01745	2,30313	0,43419
P08571	0,04245	2,2551	0,4434	0,03281	2,7823	0,3594	0,88387	0,94781	1,05506
P02652	0,03624	2,7056	0,3696	0,03247	3,3625	0,2974	0,69266	1,23700	0,80841
P30086	0,03568	1,8783	0,5324	0,08110	1,5742	0,6353	0,41714	1,24245	0,80486
P17612	0,01123	3,4230	0,2921	0,01737	3,5329	0,2831	0,05835	2,35147	0,42527
P01860	0,01622	1,9486	0,5132	0,01370	2,0003	0,4999	0,03336	2,04413	0,48921
Q5VTE0	0,00174	2,8852	0,3466	0,00611	2,7129	0,3686	0,01398	2,35331	0,42493
Q9NTK5	0,00540	9,0976	0,1099	0,05524	2,9147	0,3431	0,02276	4,57438	0,21861
P62826	0,01259	7,8279	0,1277	0,02133	8,4856	0,1178	0,07584	2,97366	0,33629
O00299	0,00060	3,9351	0,2541	0,01316	2,0831	0,4800	0,06744	1,63867	0,61025
P38606	0,00137	2,9531	0,3386	0,00760	2,4614	0,4063	0,00284	2,93126	0,34115
P31948	0,03316	3,2562	0,3071	0,23241	1,6688	0,5992	0,22847	1,74199	0,57406
Q15366	0,03144	2,1815	0,4584	0,01987	3,0474	0,3282	0,05697	2,20238	0,45405
P61978	0,00455	1,9658	0,5087	0,01090	1,7219	0,5807	0,02331	1,58640	0,63036
P09651	0,03792	1,7249	0,5798	0,10181	1,6196	0,6174	0,10803	1,53087	0,65322
P26583	0,00430	5,3391	0,1873	0,06362	2,4046	0,4159	0,21889	1,59645	0,62639
P40199	0,04332	3,9420	0,2537	0,19983	2,0064	0,4984	0,67448	1,35539	0,73780
Q92882	0,02453	3,3752	0,2963	0,26182	1,5883	0,6296	0,81802	1,09957	0,90945
